# Social frailty predicts worse outcomes in patients with multiple myeloma: A novelty in an old approach

**DOI:** 10.1002/jha2.40

**Published:** 2020-06-17

**Authors:** Toshiki Terao, Takafumi Tsushima, Daisuke Miura, Kentaro Narita, Masami Takeuchi, Kosei Matsue

**Affiliations:** ^1^ Department of Internal Medicine Division of Hematology/Oncology Kameda Medical Center Chiba Japan

**Keywords:** International Myeloma Working Group, International Staging System, multiple myeloma, physical frailty, social frailty

## Abstract

Social frailty, defined as the loss of social roles and networks in the community, has never been evaluated in patients with multiple myeloma (MM). This study aimed to evaluate the usefulness of social frailty as a predictor of survival in MM. We retrospectively reviewed 237 consecutive patients with MM from 2009 to 2019. Activities of daily living (ADL), the instrumental ADL score, the Charlson Comorbidity Index, and factors to evaluate social relationships were routinely assessed at the time of initial diagnosis and first hospitalization at our center by hematological clinicians, nurses, and rehabilitation staff. Social frailty was evaluated using five social factors and was defined as a score of at least 2 points. Overall, 69 (30.0%) patients were defined as socially frail, with a median score of 0. Those who were socially frail showed significantly shorter progression‐free and overall survival than those who were not. Using the International Staging System, International Myeloma Working Group frailty score, and social frailty, we developed two staging systems, and these further demonstrated the importance of assessing frail patients with MM. Our findings have identified the usefulness for evaluating social frailty; however, to confirm our results, an independent study with larger patient numbers with an entirely prospective assessment is needed to confirm their results.

## 1 INTRODUCTION

Novel treatment modalities have improved the prognosis of patients with multiple myeloma (MM) in the last two decades [1]. However, considering that the median age at diagnosis is the early 70s [2], more than half of patients with MM are not eligible for intensive chemotherapy and other treatments including autologous hematopoietic stem‐cell transplantation (ASCT). For elderly patients, the primary goal of treatment is the control of myeloma and maintenance of the patient's performance status (PS) [3, 4, 5]. However, treatment cannot be determined based only on patient age. Evaluation of physical function or frailty, also known as “staging the aging” [6], which includes assessments of the activities of daily living (ADL), instrumental ADL (IADL), Charlson Comorbidity Index (CCI), and myeloma frailty score recommended by the International Myeloma Working Group (IMWG), is desired [7, 8, 9, 10, 11]. Facon et al also recently found that the Eastern Cooperative Oncology Group performance status (ECOG PS), age, and CCI are useful predictors of survival among patients with transplant‐ineligible MM [12]. The Revised Myeloma Comorbidity Index (R‐MCI), developed by Engelhardt et al to weight the Initial Myeloma Comorbidity Index (I‐MCI), showed a significant survival impact when 13 risk factors including organ function and MM‐specific risks were considered [13]. Although these representative variables could predict patient outcomes and help identify patients who will benefit from triplet/quadruplet combination therapy or ASCT, these have not been used to evaluate a patient's relationship or role in the social community because the variables mainly assess physical ability, physical frailty, and organ function [4, 11, 13, 14, 15, 16, 17].

Only recently has the concept of “social frailty” been proposed [18, 19, 20]. Social frailty, referred to as social isolation, indicates the loss of social roles and networks in the community and is related to physical frailty or vulnerability [20, 21, 22, 23]. In general, elderly people become physically frail and considering the median age of 70 years at MM diagnosis, some patients may experience a decline in physical function and social relationships. The natural history and unique biology of an individual's myeloma disease presentation, comorbidities, and social environment are all relevant factors [24]. Although numerous reports regarding the biology of myeloma, both in vivo and in vitro, and physical frailty have been reported, social relationships or social frailty have never been evaluated objectively. Hence, this study aimed to evaluate social relationships and their prognostic impact in patients with MM. We hypothesized that aside from the established prognostic markers, the prognosis of patients with MM is also associated with social relationships.

## 2 METHODS

### 2.1 Study design and patients

We conducted a retrospective review of 237 consecutive patients newly diagnosed with symptomatic MM between January 2009 and December 2019 at Kameda Medical Centre, Kamogawa, Japan. Patients with primary plasma cell leukemia were excluded. Data on the patients’ clinicodemographic characteristics and outcomes were obtained from the electronic medical records. Diagnosis and treatment response were assessed using the IMWG criteria. High‐risk cytogenetic abnormalities (CAs) that were evaluated included del(17p), t(4;14), and t(14;16) based on interphase fluorescence in situ hybridization analysis. All participants or their family members provided written informed consent for inclusion in retrospective studies. The study was conducted according to the Declaration of Helsinki and was approved by the ethical review board of Kameda Medical Centre.

### 2.2 Assessment of frailty

We performed multivariable evaluations that considered pretreatment demographics, laboratory data, ECOG PS, ADL, IADL, CCI, International Staging System (ISS) stage, and presence of high‐risk CAs at the time of initial diagnosis and first hospitalization at our center by hematological clinicians, nurses, and rehabilitation staff as clinical practice. ADL and IADL scales were used to assess self‐care activities, household management tasks, and independence [9, 10]. The CCI was used to estimate the number and types of comorbidities [8]. The IMWG frailty score was calculated based on the combination of age, ADL, IADL, and CCI, and cutoff values (<1, ≥2) were followed as previously reported [11]. Cutoff values to predict reduced overall survival (OS) and progression‐free survival (PFS) were defined as follows: ADL (>4, ≤4), IADL (>5, ≤5), and CCI (≤1, >1) [11]. OS was calculated from the date of myeloma diagnosis until the date of any‐cause death, whereas PFS was calculated from the date of myeloma diagnosis until the date of progression, relapse, or any‐cause death determined from the electronic medical records.

To assess social frailty, five factors regarding daily social activities, roles, and relationships were evaluated as follows [20, 23]: (a) “Going out less frequently when compared with the previous year”; (b) “Sometimes visiting your friends”; (c) “Feeling that you are helpful to friends or family”; (d) “Living alone”; and (e) “Talking with someone every day.” These factors were recorded in different parts of general initial evaluations that nurses and rehabilitation staff performed at the first hospitalization. One point (maximum possible score, 0‐5) was allocated for positive responses to factors 1 and 4 or negative responses to factors 2, 3, and 5. Patients were defined as having social frailty if they scored at least 2 out of 5 points [20].

### 2.3 Statistical analysis

Baseline characteristics were compared between patients with and without social frailty using the Mann‐Whitney *U*‐tests or Student *t*‐tests for continuous variables and the Fisher exact tests for categorical variables. The probability of PFS and OS was estimated using the Kaplan‐Meier method and compared using the log‐rank test. The prognostic impact of social frailty was evaluated using univariate and multivariate Cox proportional hazards analyses. Variables that were associated with social frailty or had *P*‐values < 0.1 in univariate analysis were further tested in the multivariate analysis. All statistical analyses were conducted using EZR software (Saitama Medical Center, Jichi Medical University), which is a graphical user interface for R version 3.1.2 (The R Foundation for Statistical Computing, Vienna, Austria) [25]. Two‐sided *P*‐values < .05 were considered statistically significant.

## 3 RESULTS

### 3.1 Patient characteristics

The baseline clinicodemographic characteristics of the patients are summarized in Table 1. The median patient age was 73 years (interquartile range [IQR]: 66‐81 years), and 113 of 237 (47.7%) patients were aged at least 75 years. The median observation period was 33.3 months (IQR: 12.5‐65.6 months). The median OS and 3‐year OS rates were 76.2 months (95% confidence index [CI], 59.2‐113.1) and 73.3% (95% CI, 66.3‐79.0%), respectively. Overall, 87 patients (36.7%) died during the study period. The number of patients with Revised ISS (R‐ISS) stages I, II, and III was 31 (13.1%), 155 (65.4%), and 51 (21.5%), respectively. Overall, 52 patients (21.9%) had high‐risk CAs. The median ADL, IADL, CCI, and IMWG were 5 (IQR: 4‐5), 5 (IQR: 2‐8), 1 (IQR: 0‐2), and 2 (IQR: 0‐3), respectively. In addition, 87 patients (36.7%) had a PS score of ≥3; 77 (32.5%), an ADL score of ≤4; 121 (51.1%), an IADL score of ≤5; and 75 (31.6%), a CCI of ≥2. The median IMWG frailty score was 2 (0‐3), and 132 patients (55.7%) were identified as being physically frail.

**TABLE 1:** Patients characteristics in the overall cohort

Characteristics	All patients	Not social frailty	Social frailty^a^	*P‐*value
	n = 237	n = 161	n = 69	
Age, years [median (IQR)]	73.0 (66.0, 81.0)	72.0 (65.0, 79.0)	77.0 (69.0, 83.0)	.005
Sex, male (%)	122 (51.5)	77 (47.8)	33 (47.8)	1
Albumin, mg/dL [median (IQR)]	3.2 (2.7, 3.8)	3.4 (2.8, 3.9)	2.9 (2.4, 3.3)	<.001
Beta 2‐microglobulin, mg/L [median (IQR)]	4.9 (2.9, 8.4)	4.3 (2.6, 7.5)	6.6 (4.0, 10.4)	<.001
Calcium, mg/dL [median (IQR)]	9.8 (9.3, 10.5)	9.6 (9.2, 10.3)	10.2 (9.6, 11.1)	<.001
Creatinine, mg/dL [median (IQR)]	0.9 (0.7, 1.5)	0.9 (0.7, 1.5)	1.0 (0.8, 1.5)	.293
Hemoglobin, g/dL [median (IQR)]	9.5 (8.3, 11.3)	9.9 (8.3, 11.5)	9.3 (8.2, 10.4)	.112
LDH, high (%)	82 (34.6)	59 (36.6)	20 (29.0)	.291
High‐risk CAs	52 (21.9)	35 (21.7)	16 (23.2)	.863
ISS (%)				
Stage I	46 (19.4)	42 (26.1)	3 (4.3)	<.001
Stage II	80 (33.8)	52 (32.3)	25 (36.2)	
Stage III	111 (46.8)	67 (41.6)	41 (59.4)	
R‐ISS (%)				
Stage I	31 (13.1)	29 (18.0)	1 (1.4)	<.001
Stage II	155 (65.4)	102 (63.3)	49 (71.0)	
Stage III	51 (21.5)	30 (18.6)	19 (27.5)	
PS, score [median (IQR)]	1 (1, 3)	1 (0, 2)	3 (2, 4)	<.001
PS ≥ 3 (%)	87 (36.7)	14 (23.3)	27 (71.1)	<.001
ADL, score [median (IQR)]	5 (4, 5)	5 (5, 5)	3 (1, 5)	<.001
ADL ≤ 4 (%)	77 (32.5)	26 (16.1)	48 (69.6)	<.001
IADL, score [median (IQR)]	5 (2, 8)	7 (4, 8)	2 (0, 3)	<.001
IADL ≤ 5 (%)	121 (51.1)	52 (32.3)	63 (91.3)	<.001
CCI, score [median (IQR)]	1 (0, 2)	1 (0, 1)	1 (0, 3)	<.001
CCI ≥ 2 (%)	75 (31.6)	39 (24.2)	34 (49.3)	<.001
IMWG frailty, score [median (IQR)]	2 (0, 3)	1 (0, 2)	3 (2, 4)	<.001
IMWG frailty ≥2 (%)	132 (55.7)	62 (38.5)	63 (91.3)	<.001
Social frailty, score [median (IQR)]	0 (0, 2)	–	–	–
Factors for social frailty (%)^a^				
Going out less frequency within last year (positive)	69 (30.0)	18 (11.2)	51 (73.9)	<.001
Visiting friends sometimes (negative)	69 (30.0)	9 (5.6)	60 (87.0)	<.001
Feeling helpful to friends or family (negative)	50 (21.7)	4 (2.5)	46 (66.7)	<.001
Living alone (positive)	23 (10.0)	8 (5.0)	15 (21.7)	<.001
Talking with someone everyday (negative)	9 (3.9)	0 (0.0)	9 (13.2)	<.001
Induction regimen (%)^b^				
Doublet	96 (41.2)	52 (32.3)	39 (56.5)	.001
Triplet	133 (57.1)	105 (65.2)	26 (37.7)	<.001
With PI	223 (95.7)	154 (95.7)	63 (91.3)	.217
With IMiDs	60 (25.8)	47 (29.2)	12 (17.4)	.07
Received ASCT (%)	65 (27.9)	52 (32.3)	13 (18.8)	.039

Abbreviations: ADL, activities of daily living; ASCT, autologous hematopoietic stem‐cell transplantation; CA, chromosomal abnormality; CCI, Charlson Comorbidity Index; IADL, instrumental activities of daily living; IMiDs, immunomodulatory drugs; IMWG, International Myeloma Working Group; ISS, International Staging Score; LDH, lactate dehydrogenase; PI, proteasome inhibitor; PS, performance status; R‐ISS, Revised International Staging Score.

a n = 230 (seven patients could not be evaluated due to loss of data).

b n = 233 (four patients could not receive induction therapy).

### 3.2 Assessment of social frailty

Seven patients (3.0%) were excluded in the analysis of social frailty because the induction regimen was initiated as an outpatient procedure without any hospitalization. Of the remaining 230 patients, 69 (30.0%) were going out less frequently than that in the previous year (positive = 1 point), 69 (30.0%) were visiting friends sometimes (negative = 1), 50 (21.7%) were feeling helpful to friends or family (negative = 1), 23 (10.0%) were living alone (positive = 1), and nine (3.9%) were talking with someone every day (negative = 1). The median social frailty score was 0 out of 5 (IQR: 0‐2), and 69 patients (30.0%) were defined as being socially frail (Table 1). Patients with social frailty showed significantly worse ADL, IADL, CCI, PS, and IMWG frailty scores than did those without social frailty (median ADL: 5 vs 3; IADL: 7 vs 2; CCI: 1 vs 1; PS: 1 vs 3; IMWG frailty score: 1 vs 3; *P* < .001 for all). Moreover, the levels of biological markers, including albumin, creatinine, and hemoglobin (Hb) and myeloma status including lactate dehydrogenase, beta 2‐microglobulin (B2M), ISS, and R‐ISS were relatively lower in the patients with social frailty than those without social frailty (Table 1). Patients who were administered doublet induction chemotherapy were more socially frail (39, 56.5%) than those administered triplet induction chemotherapy (52, 32.3%). There were no significant differences in the use of either proteasome inhibitors or immunomodulatory drugs in the induction chemotherapy regimens between patients with and without social frailty. Significantly fewer patients in the socially frail group had ASCT (52 [32.3%] vs 13 [18.8%], *P* = .039).

### 3.3 Prediction of OS and PFS evaluated via IMWG frailty score and social frailty

**FIGURE 1:**
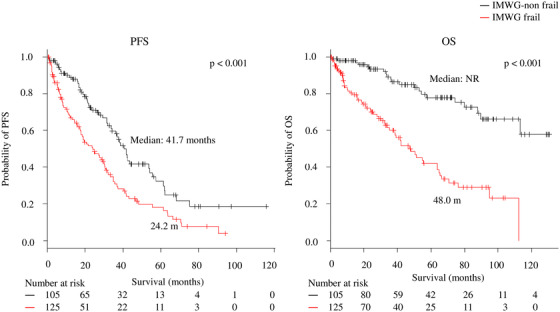
Progression‐free survival (PFS) and overall survival (OS) according to physical frailty by International Myeloma Working Group (IMWG) frailty scores *Note*. Median PFS: 24.2 versus 41.7 months, *P* < .001; median OS: 48 months versus not reached, *P* < .001

**FIGURE 2:**
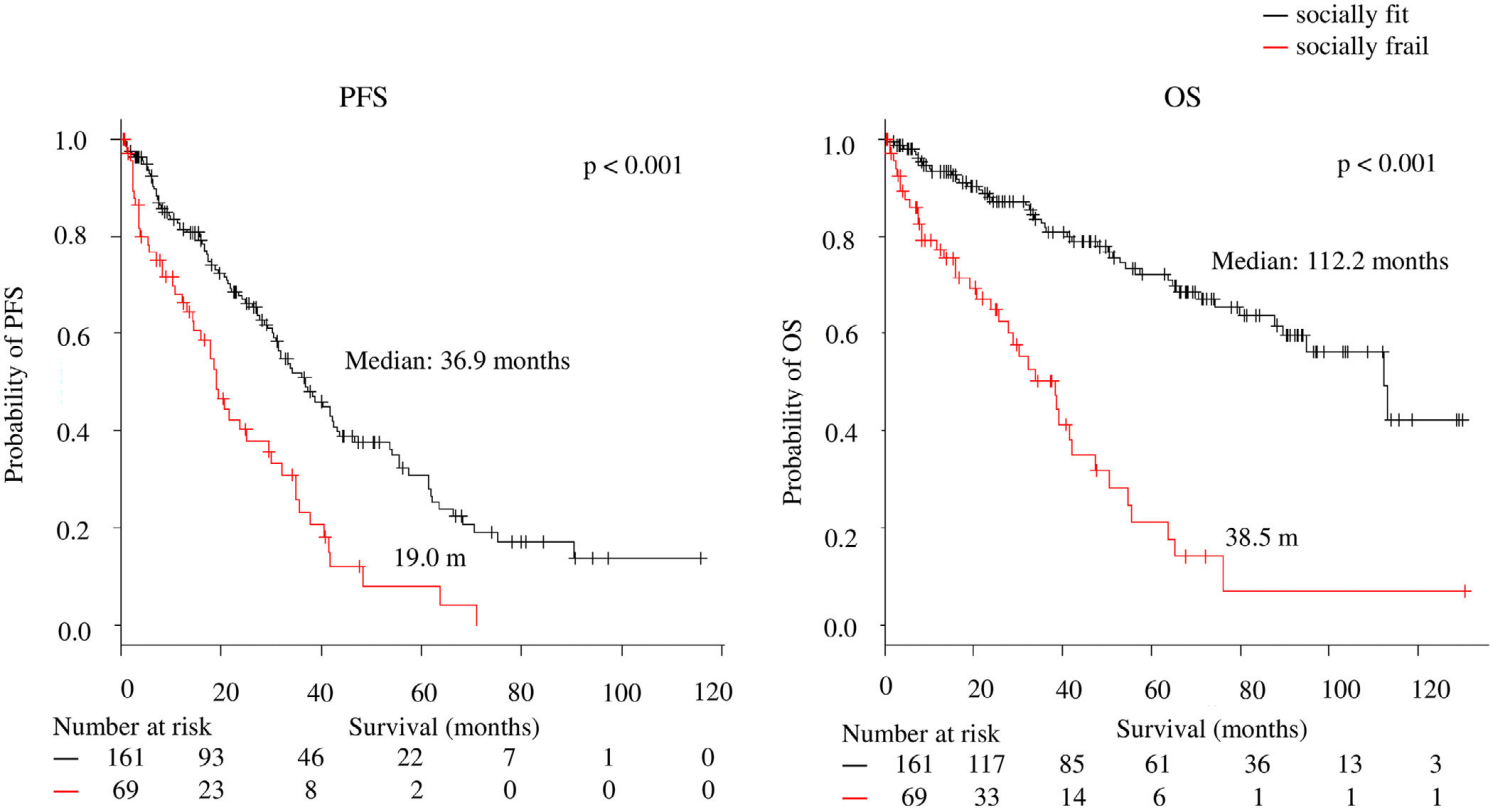
Progression‐free survival (PFS) and overall survival (OS) according to social frailty *Note*. Median PFS: 19.0 versus 36.9 months, *P* < .001; median OS: 38.5 versus 112.2 months, *P* < .001

The PFS (median: 24.2 months vs 41.7 months; *P* < .001) and OS (median: 48 months vs not reached [NR]; *P* < .001) were shorter in the patients with IMWG frailty than those without IMWG frailty (Figure 1). In addition, socially frail patients had a significantly shorter PFS and OS than those who were not socially frail (median PFS: 19.0 vs 36.9 months, respectively, *P* < .001; median OS: 38.5 vs 112.2 months, respectively, *P* < .001; Figure 2).

### 3.4 Prediction of OS and PFS evaluated via combination of ISS, IMWG frailty score, and social frailty

**FIGURE 3:**
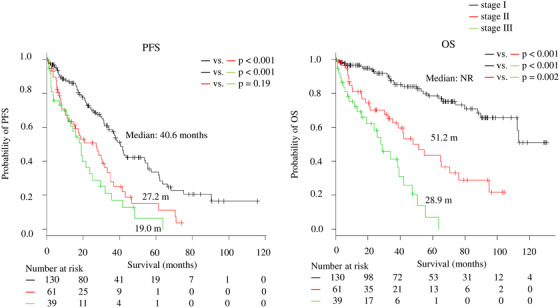
Progression‐free survival (PFS) and overall survival (OS) according to the Disease‐Physical‐Social staging system *Note*. The patients were divided into three groups according to the presence of International Staging System (ISS) stage III, International Myeloma Working Group (IMWG) frailty, and social frailty, as follows: stage I, II, and III included patients with zero or one, two, and all three of the parameters, respectively. Median PFS: 40.6, 27.2, and 19.0 months for stages I, II, and III, respectively; *P* < .001, < .001, and .19 for stage I versus II, stage I versus III, and stage II versus III, respectively. Median OS: not reached (NR), 51.2, and 28.9 months for stages I, II, and III, respectively; *P* < .001, < .001, and .002 for stage I versus II, stage I versus III, and stage II versus III, respectively

As the assessment of frailty scores and myeloma disease status is important, we combined scores that assessed ISS stage III, IMWG‐frailty, and social frailty (Disease‐Physical‐Social staging system) and divided the patients into three groups: stage I, II, and III included patients with zero or one, two, and all three of the parameters, respectively. There were significant differences in PFS (median: 40.6, 27.2, and 19.0 months for stages I, II, and III, respectively; *P* < .001, < .001, and .19 for stage I vs II, stage I vs III, and stage II vs III; respectively) and OS (median: NR, 51.2, and 28.9 months for stages I, II, and III, respectively; *P* < .001, < .001, and .002 for stage I vs II, stage I vs III, and stage II vs III, respectively) among the three groups (Figure 3).

**FIGURE 4:**
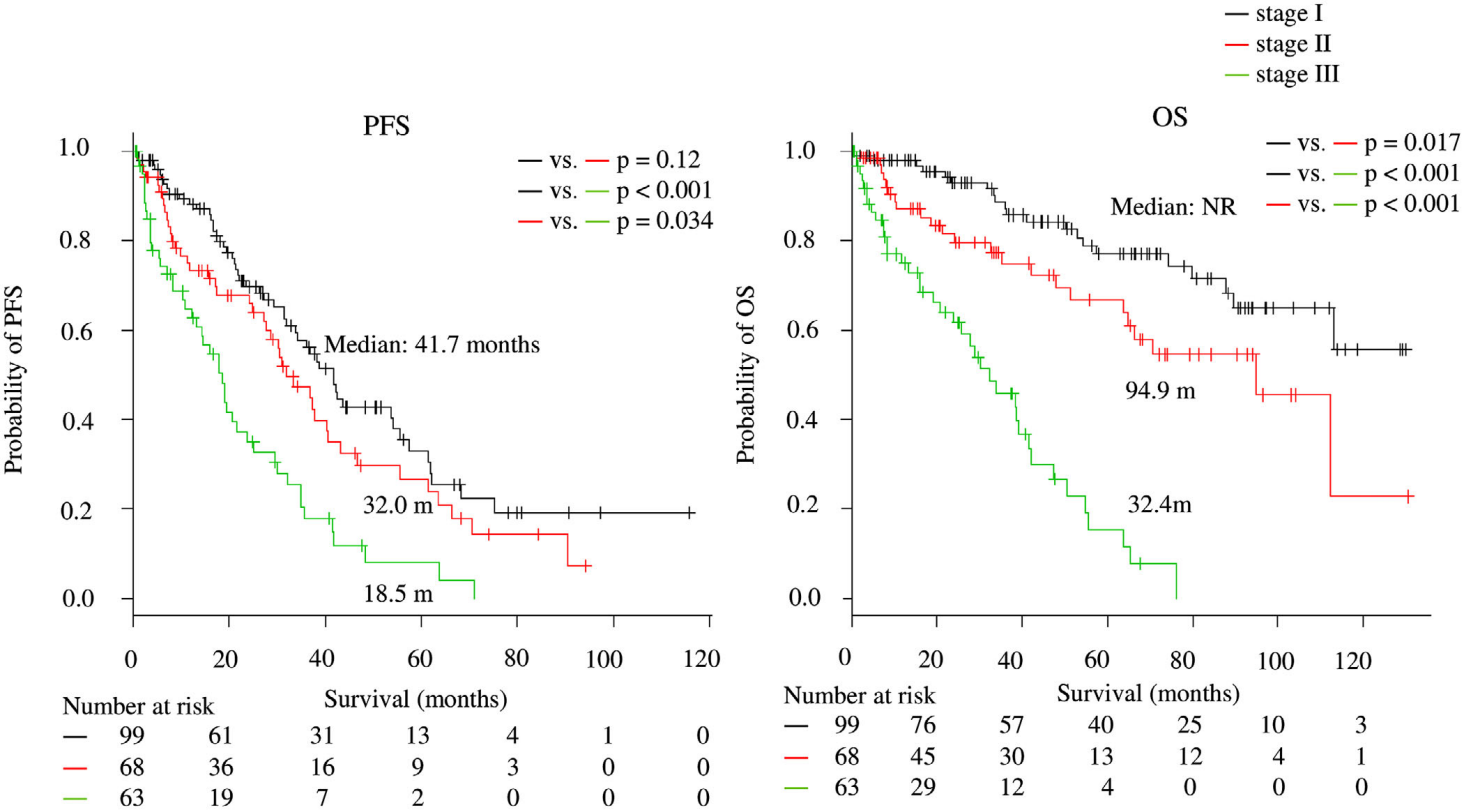
Progression‐free survival (PFS) and overall survival (OS) according to the Physical‐Social Frailty staging system *Note*. The patients were divided into three groups according to the presence of International Myeloma Working Group (IMWG) frailty and social frailty, as follows: stage I, II, and III included patients with zero, one, and all two of the parameters, respectively. Median PFS: 41.7, 32.0, and 18.5 months for stages I, II, and III, respectively; *P* = .12, < .001, and .034 for stage I versus II, stage I versus III, and stage II versus III, respectively. Median OS: not reached (NR), 94.9, and 32.4 months for stages I, II, and III, respectively; *P* = .017, < .001, and < .001 for stage I versus II, stage I versus III, and stage II versus III, respectively

Moreover, to show the importance of evaluating frailty, we combined other scores that assessed IMWG‐based frailty and social frailty (Physical‐Social Frailty staging system) and divided the patients into three groups: stage I, II, and III included patients with zero, one, and all two of the parameters, respectively. There were also significant differences in PFS (median: 41.7, 32.0, and 18.5 months for stages I, II, and III, respectively; *P* = .12, < .001, and .034 for stage I vs II, stage I vs III, and stage II vs III, respectively) and OS (median: NR, 94.9, and 32.4 months for stages I, II, and III, respectively; *P* = .017, < .001, and < .001 for stage I vs II, stage I vs III, and stage II vs III, respectively) (Figure 4).

**FIGURE 5:**
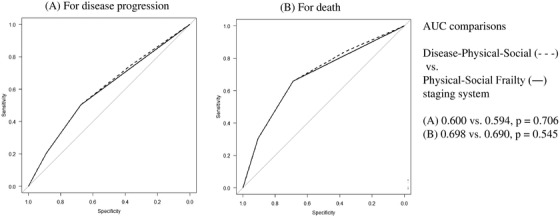
Comparisons of the area under the receiver operating characteristics (ROC) curves of each prognostic system used to predict disease progression and death *Note*. Comparisons between Disease‐Physical‐Social and Physical‐Social Frailty staging systems for (A) disease progression and (B) death. Area under the curve (AUC) for predicting progression: .60 versus .594, *P* = .706; AUC for predicting death: .698 versus .69, *P* = .545

Receiver operating characteristics (ROC) curves were developed to compare the two staging‐systems: the Disease‐Physical‐Social system and the Physical‐Social Frailty staging system. The area under the curve was extremely similar as both predicted disease progression and death (predicting progression: 0.60 vs. 0.594, *p* = 0.706; death: 0.698 vs. 0.69, *p* = 0.545, Figure 5).

### 3.5 Univariate and multivariate analyses of social frailty scores

Finally, to evaluate the usefulness of social frailty as a prognostic factor, we conducted uni‐ and multivariate analyses. In the univariate analysis, older age (>74 years), calcium ≥ 11.5 mg/dL, creatinine ≥ 2.0 mg/dL, Hb ≤ 10.0 g/dL, B2M ≥ 5.5 mg/L, high‐risk CAs, ISS stage III, R‐ISS stage III, worse PS (≥3), worse ADL (≤4), worse IADL (≥5), worse CCI (≥2), IMWG‐frailty, treatment regimen (doublet or triplet), and social frailty were significantly associated with prognosis (Table 2). In the multivariate analysis, social frailty remained a significant prognostic factor even after adjustment for older age, hemoglobin, LDH, high‐risk CAs, ISS stage III, IMWG‐frailty, and triplet regimen (Table 3).

**TABLE 2:** Univariate analysis of the factors predictive of progression‐free survival (PFS) and overall survival (OS)

	PFS		OS	
Variables	HR (95% CI)	*P*‐value	HR (95% CI)	*P*‐value
Age ≥ 75 years	1.45 (1.04‐2.02)	.029	1.92 (1.24‐2.98)	.003
Calcium ≥ 11.5 mg/dL	1.43 (0.98‐2.10)	.063	1.38 (0.85‐2.24)	.19
Creatinine ≥ 2.0 mg/dL	1.55 (1.10‐2.18)	.013	1.36 (0.87‐2.13)	.18
Hemoglobin ≤ 10.0 g/dL	1.39 (1.00‐1.94)	.052	1.72 (1.11‐2.68)	.016
Beta 2‐microglobulin ≥ 5.5 mg/L	1.52 (1.08‐2.14)	.015	1.80 (1.13‐2.88)	.014
LDH, high	1.03 (0.73‐1.46)	.874	1.04 (0.66‐1.64)	.859
High‐risk CAs	1.54 (1.05‐2.27)	.029	1.09 (0.65‐1.83)	.731
ISS, Stage III^a^	1.61 (1.15‐2.26)	.005	2.20 (1.41‐3.44)	<.001
R‐ISS, Stage III^b^	1.41 (0.95‐2.11)	.092	1.56 (0.95‐2.56)	.082
PS ≥ 3	1.43 (1.01‐2.02)	.044	2.31 (1.48‐3.60)	<.001
ADL ≤ 4	1.45 (1.03‐2.06)	.035	2.66 (1.71‐4.14)	<.001
IADL ≤ 5	1.75 (1.25‐2.50)	.001	3.47 (2.16‐5.60)	<.001
CCI ≥ 2	1.48 (1.05‐2.09)	.025	1.97 (1.27‐3.05)	.003
IMWG frailty ≥ 2	1.93 (1.37‐2.71)	<.001	4.14 (2.48‐6.92)	<.001
Doublet regimen	1.32 (0.95‐1.84)	.101	1.52 (0.98‐2.35)	.059
Triplet regimen	0.73 (0.52‐1.02)	.063	0.57 (0.37‐0.88)	.012
Social frailty score ≥ 2	2.19 (1.54‐3.14)	<.001	4.24 (2.70‐6.67)	<.001

Abbreviations: ADL, activities of daily living; CAs, cytogenetic abnormalities; CCI, Charlson Comorbidity Index; CI, confidence interval; HR, hazard ratio; IADL, instrumental activities of daily living; IMWG, International Myeloma Working Group; ISS, International Staging Score; LDH, lactate dehydrogenase; OS, overall survival; PFS, progression‐free survival; PS, performance status; R‐ISS, Revised International Staging Score.

a Referred to ISS stage I.

b Referred to R‐ISS stage I.

**TABLE 3:** Multivariate analysis of the factors predictive of PFS and OS

	PFS		OS	
Variables	HR (95% CI)	*P*‐value	HR (95% CI)	*P*‐value
Age ≥ 75 years	1.15 (0.76‐1.75)	.507	1.21 (0.73‐2.00)	.462
Hemoglobin ≤ 10.0 g/dL	1.11 (0.77‐1.61)	.576	1.18 (0.71‐1.94)	.524
LDH, high	1.28 (0.89‐1.84)	.180	1.42 (0.89‐2.26)	.142
High‐risk CAs	1.62 (1.10‐2.39)	.015	1.18 (0.70‐2.01)	.534
ISS, stage III^a^	1.32 (0.93‐1.87)	.123	1.69 (1.06‐2.68)	.026
IMWG frailty ≥ 2	1.62 (1.11‐2.37)	.013	2.57 (1.45‐4.55)	.001
Triplet regimen	0.95 (0.66‐1.35)	.759	0.92 (0.58‐1.45)	.713
Social frailty score ≥ 2	1.74 (1.17‐2.59)	.007	2.84 (1.73‐4.67)	<.001

Abbreviations: CI, confidence interval; HR, hazard ratio; IMWG, International Myeloma Working Group; ISS, International Staging Score; LDH, lactate dehydrogenase; OS, overall survival; PFS, progression‐free survival; PS, performance status.

a Referred to ISS stage I.

## 4 DISCUSSION

This study demonstrated the prognostic impact of social frailty in patients with MM, in addition to the established prognostic markers. Several studies have shown the negative impact of frailty using the physical conditions of patients or the presence of comorbidities as proxy measures [11, 12, 13]. However, to our best knowledge, this is the first study to evaluate and emphasize on social frailty in the assessment of patients with MM.

There have been several studies on social frailty, and thus far, five factors can be used for the objective evaluation of social relationships. However, these five factors have different impact in patients with MM. The first factor “Going out less frequently when compared with the previous year” may be influenced by pain associated with bone lesions and is therefore considered important in the assessment of patients with MM. Patients with high scores in this factor showed significantly higher hypercalcemia and lower PS in our analysis (data not shown). However, only a small number of patients (nine of 230 patients) had high scores for factor 5 (ie, “Talking with someone every day”), and all the nine patients were included in the social frailty group. This factor may not be essential to know the social frailty in MM because few numbers of patients who were scored had less possibility to contribute to the judgement of social frailty. The prognostic impact of social frailty is already known in various patients [20, 26, 27, 28]. However, it would be necessary to determine more appropriate factors about social frailty for patients with MM. Moreover, assessing social frailty at different time points (eg, at initial diagnosis, at relapse when patients need second line therapy, or at the time of ASCT) may help determine when patients need social intervention to help prevent further frailty.

There are also numerous reports regarding physical frailty [11, 13, 14, 15]. The IMWG frailty score, which we used in this study, was devised based on data of patients included in clinical trials and was therefore reported to be slightly dissociated from real‐world data [14]. We evaluated consecutive MM patients, and excluded only seven patients, and although the number of patients was relatively small to reflect real‐world data, the findings were from a clinical setting. Our results on physical fragility based on ADL, IADL, and CCI were worse than those of a previous report on IMWG [11] but consistent with that of a previous prospective study [14].

By combining scores on ISS stage III, physical frailty, and social frailty, we were able to stratify the prognosis of patients with MM (Disease‐Physical‐Social staging system). In addition, we devised another staging system by combining physical and social frailty to create a simplified evaluation tool and to emphasize the importance of frailty in MM (Physical‐Social Frailty staging system). ROC curve analysis showed no significant difference in the predictive capability for PFS or OS between the two staging systems. In addition, the Physical‐Social Frailty staging system enabled a comprehensive evaluation of frailty, thereby emphasizing the importance of evaluating the frailty in patients with MM.

This study has some limitations despite the strength of evaluating social frailty. First, and importantly, the study's retrospective nature might have introduced biases (eg, information bias) and thus social frailty maybe over or underscored. However, the factors for both physical and social frailty were already assessed by medical staff during the first hospitalization, thus minimizing the influence of such bias in this study. Second, the organ function of patients, such as lung and renal function, included in the assessment of major physical frailty scores (I‐/R‐MCI) were not evaluated [14]. Third, this study was limited by its retrospective nature and heterogeneous treatments. It was conducted at a single institution, and the findings were not validated. Fourth, at our institute, there were no caregivers designated by a specific team (eg, bone marrow transplantation team) [29, 30, 31], and it was not possible to evaluate the influence of caregivers objectively and separately. Finally, no absolute definition or established evaluation methods for social frailty have been identified. Despite these limitations, this study identified a novel approach for evaluating social frailty in patients with MM. Prospective studies with a larger sample size are needed to confirm our findings.

In summary, we stratified patients with MM based on their prognosis using a simple social assessment and proposed a new prognostic staging system. Albeit the retrospective review and several limitations, this report identified a novel approach for evaluating social frailty in patients with MM. To confirm our results, an independent study with larger patient numbers with an entirely prospective assessment is needed.

## AUTHOR CONTRIBUTIONS

TT conceived, designed, and initiated the study, collected the data, performed statistical analysis, wrote the manuscript, and provided patient care. TT, DM, KN, and MT provided patient care. KM supervised the study, collected the data, wrote the manuscript, and provided patient care. All authors have reviewed and approved the final manuscript.

## CONFLICT OF INTEREST

The authors declare no conflict of interest.
